# Análise de concordância em estudos clínicos e experimentais

**DOI:** 10.1590/1677-5449.004216

**Published:** 2016

**Authors:** Hélio Amante Miot

**Affiliations:** 1 Universidade Estadual Paulista – UNESP, Faculdade de Medicina de Botucatu, Departamento de Dermatologia e Radioterapia, Botucatu, SP, Brasil.

Análise de concordância se refere à capacidade de aferir resultados idênticos (mesma unidade de medida), aplicados ao mesmo sujeito/fenômeno, quer por instrumentos diferentes, pelo mesmo instrumento em tempos diferentes, por avaliadores diferentes, ou por alguma combinação dessas situações. Exemplos triviais são calibragem de instrumentos, fidedignidade de escala/medida, avaliação de equivalência entre ferramentas de mensuração, julgamento em provas de habilidades, avaliação de repetitividade ou reprodutibilidade, e análise diagnóstica (concordância interpessoal e intrapessoal) e psicométrica (estabilidade temporal)^1^^,^ 2.

Com frequência, demandas de análise de concordância são avaliadas, erroneamente, por técnicas estatísticas de correlação (por exemplo, coeficiente de Pearson), que pressupõem apenas que a variação dos valores de uma variável acompanhe a variação dos valores de outra. No entanto, para a análise de concordância, além de correlação, deve haver coincidência entre os valores. Por essa razão, as medidas de efeito de concordância costumam ser menores que os coeficientes de correlação, quando aplicadas ao mesmo conjunto de dados^3^^-^ 5.

A definição do modelo analítico de concordância deve ser idealizada precocemente, na elaboração do projeto, de forma que seja contemplado um desenho que favoreça a coleta, a análise e a interpretação de dados. Nessa fase, contatar um estatístico experiente aumenta a chance de sucesso.

Em princípio, a análise de concordância pode depender unicamente da definição predeterminada do pesquisador, que deve definir um limite tolerável para satisfazer suas necessidades. Isso ocorre comumente em calibragem e equivalência de ferramentas de mensuração, nas quais as aferições devem obedecer a uma variação percentual máxima em comparação a uma medida-padrão ou um instrumento específico. Entretanto, a existência inerente de erro aleatório de medidas ligada ao instrumento e/ou aos avaliadores inclui uma variação intrínseca das medidas, que interfere na estimativa de concordância. Para avaliar esses aspectos, foram desenvolvidos diversos testes estatísticos específicos, e os principais serão discutidos a seguir.

A situação mais simples ocorre quando a variável de interesse é dicotômica (por exemplo, doente × saudável, indicação cirúrgica × clínica, aprovado × reprovado), e a estimativa ocorre por dois avaliadores ou dois instrumentos; nesse caso, classicamente se emprega a estatística *kappa* de Cohen. O valor, o intervalo de confiança e a significância estatística de *kappa* devem ser interpretados como a dimensão da concordância que ultrapassa a coincidência de avaliações que ocorrem ao acaso^6^.

Como exemplo, a investigação de Barros et al.^7^ empregou Doppler dos membros inferiores *versus* transvaginal para a identificação de varizes pélvicas (Tabela 1), evidenciando concordância total de (62+93)/249 = 62,2%. Os 94 (37,8%) casos discordantes se distribuíram de forma bastante assimétrica, o que revelou maior falha diagnóstica do exame dos membros inferiores. O coeficiente *kappa* resultou em fraca concordância – 0,31 (IC95% 0,20-0,40), p < 0,01 –, apesar de estatisticamente significativa.

**Tabela 1:** Evidência de varizes pélvicas pela ultrassonografia com Doppler dos membros inferiores *versus* método transvaginal (n = 249)^7^.

**Membros inferiores**	**Transvaginal**		**Total**
	**Positivo**	**Negativo**	
**Positivo**	62	6	68
**Negativo**	88	93	181
**Total**	150	99	249

Uma situação mais elaborada ocorre quando uma variável ordinal (por exemplo, estágios de doença, níveis de gravidade, estimativa em “cruzes”, acerto total x parcial x erro) é estimada por dois avaliadores. Nesse caso, além das concordâncias totais, um peso pode ser atribuído para classificações próximas, em detrimento das maiores divergências. Para essa análise, utiliza-se, classicamente, a estatística *kappa* com pesos quadráticos (Fleiss-Cohen)^6^^,^ 8.

Quando a mesma amostra é analisada, o estimador *kappa* com pesos apresenta maior magnitude que a medida de concordância completa, por incorporar o conceito de concordância parcial. Há uma variedade de formas de estabelecer pesos para as concordâncias parciais. Usualmente, *kappa* com pesos quadráticos apresenta o mesmo resultado do coeficiente de correlação intraclasse (CCI), discutido adiante^8^^,^ 9.

Por exemplo, utilizando os dados brutos do trabalho de Brianezi et al.^10^, em que dois pesquisadores classificaram a marcação epidérmica imuno-histoquímica da proteína p53 a partir de uma escala ordinal de zero a quatro “cruzes”, observou-se pobre concordância total (16/63=25,4%) entre os avaliadores (Tabela 2). Entretanto, o coeficiente *kappa* com pesos resultou em uma concordância substancial – 0,66 (0,40-0,87) –, devido ao fato de que o avaliador 1 classificara sistematicamente as imagens em um nível maior que o avaliador 2, gerando alta concordância parcial. Outrossim, quando se dispõem de múltiplos níveis ordinais, as classificações dos valores extremos (por exemplo, 0 ou 4+) costumam resultar em maior concordância do que as categorias intermediárias.

**Tabela 2:** Avaliação comparativa da marcação (0 a 4+) epidérmica imuno-histoquímica da proteína p53 por dois pesquisadores experientes (n = 63)^10^.

		**Av2**					**Total**
		**0**	**1+**	**2+**	**3+**	**4+**	
Av1	0	10	-	-	-	-	10
	1+	12	2	-	-	-	14
	2+	7	8	1	-	-	16
	3+	-	6	4	1	-	11
	4+	-	-	3	5	4	12
Total		29	16	8	6	4	63

Av1: avaliador 1; Av2: avaliador 2.

Quando as variáveis de interesse são quantitativas (discretas, contínuas ou *ranks*) e ocorre a estimativa por dois avaliadores (interobservador), dois instrumentos ou as variáveis são estimadas em momentos diferentes (teste-reteste), emprega-se comumente o CCI para completa concordância, que é robusto inclusive para violações de normalidade das distribuições^11^^-^ 14. Há diferentes algoritmos de cálculo do CCI para avaliar correlação e concordância. Porém, neste texto, importam os algoritmos de completa concordância. Entre esses, o pesquisador deve optar por: aleatório de uma via, aleatório de duas vias ou misto de duas vias, de acordo com a natureza dos avaliadores. No primeiro, os avaliadores não são os mesmos para cada fenômeno avaliado; no segundo, os avaliadores são os mesmos para cada fenômeno e são escolhidos aleatoriamente (mais empregado); no terceiro, os avaliadores não são aleatórios, mas os únicos possíveis (por exemplo, análise intraobservador). Por fim, o pesquisador deve optar pelo CCI de medidas únicas, quando importa a concordância da medida de cada avaliador em relação ao comportamento dos n avaliadores (mais empregado), ou pelo CCI de medidas médias, quando o escore da variável é composto pela combinação dos n escores dos avaliadores. Essas opções podem levar a indicadores de diferentes magnitudes^15^^,^ 16.

Como exemplo, serão utilizados os dados brutos de Ianhez et al.^17^, que promoveram a contagem de lesões cutâneas múltiplas por dois avaliadores treinados – um deles, em dois momentos diferentes (A, B1 e B2) –, a fim de validar um sistema de contagem padronizado de queratoses actínicas dos membros superiores (n = 60). O CCI para concordância completa da comparação (misto de duas vias) intraobservador (B1 x B2) resultou 0,74 (0,60-0,84) para medidas únicas e 0,85 (0,75-0,91) para medidas médias. Já o CCI (aleatório de duas vias) interobservador (A x B1) resultou 0,68 (0,47-0,82) para medidas únicas e 0,81 (0,64-0,90) para medidas médias, sempre com p < 0,01. Esses resultados indicaram haver maior consistência quando um mesmo avaliador contou duas vezes as lesões, mostrando o benefício de se utilizar, como estimativa, a média de duas medidas.

Usualmente, a concordância interobservador é menor que a intraobservador para a estimativa da mesma amostra, porque incorpora variabilidades inerentes a diferentes avaliadores. Além disso, a estimativa de CCI para medidas únicas gera estimadores de menor dimensão do que a estimativa para medidas médias, o que justifica o uso de múltiplas medidas para reduzir o erro aleatório^17^.

Além da cuidadosa descrição metodológica do processo de seleção de sujeitos e avaliadores, e de coleta dos dados e das técnicas analíticas empregadas, os resultados de investigações de concordância devem ser expressos pelos dados percentuais de concordância (total e de subgrupos), além dos estimadores, com seus intervalos de confiança de 95% e sua significância estatística. Somente assim é possível interpretar em que circunstâncias as variáveis divergem. A interpretação da magnitude dos estimadores de concordância (*kappa* ou CCI) é convencionada como: 0 (ausência), 0-0,19 (pobre), 0,20-0,39 (fraca), 0,30-0,59 (moderada), 0,60-0,79 (substancial), e ≥ 0,80 (quase completa)^4^^,^ 6 ^,^ 16.

Há generalizações dos algoritmos de cálculo de *kappa* e do CCI para múltiplas avaliações, assim como diferentes combinações de sujeitos e avaliadores. Entretanto, esses métodos transcendem o escopo deste texto^1^^,^ 18.

A concordância de variáveis de natureza quantitativa pode ser representada graficamente, em pares, pelo diagrama de Bland-Altman, que projeta no eixo das ordenadas a diferença absoluta das medidas de cada ponto, e, nas abscissas, sua média^2^. Além de exibir toda a distribuição, ela permite avaliar tendências de piora da concordância de acordo com a dimensão das medidas (Figura 1). Entretanto, não consiste em um bom estimador da dimensão da concordância. Por isso, são preferíveis os testes de CCI previamente citados, como complemento à representação gráfica.

**Figura 1:**
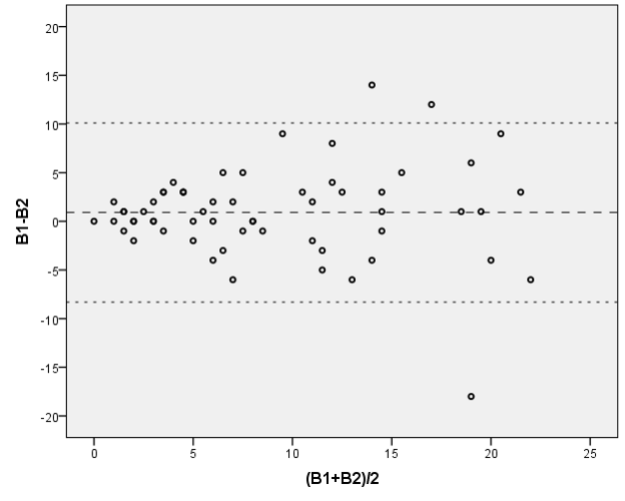
Diagrama de Bland-Altman das contagens intraobservadores (B1 x B2) de lesões cutâneas (queratoses actínicas) dos membros superiores (n = 60)^17^. Linha tracejada: média das diferenças das medidas. Linhas pontilhadas: intervalo (95%) das distribuições das diferenças das medidas.

Ainda utilizando os dados brutos de Ianhez et al.^17^, a análise da Figura 1 permite identificar maior consistência nas contagens para valores abaixo de 10 lesões. É, pois, usual que a concordância sofra efeito da dimensão das medidas. A limitação de um intervalo (por exemplo, inclusão de pacientes com menos de 10 lesões) em um estudo clínico torna os valores mais confiáveis.

O dimensionamento amostral para testes de concordância depende da dimensão de *kappa* (ou CCI), do poder do teste e da homogeneidade das categorias avaliadas. Esse assunto é adequadamente explorado em literatura específica^6^^,^ 19 ^,^ 20.

Os indicadores de concordância são influenciados pela representatividade de cada classe analisada, o que exige a máxima homogeneidade dos subgrupos, mas também pela modificação da escala original das medidas (por exemplo, transformação Log ou x^1/n^). São essenciais o treinamento prévio e o controle do rigor das estimativas dos avaliadores, porque podem resultar em discordância entre as estimativas, o que adiciona um erro sistemático, em detrimento da dimensão das medidas aferidas^1^^,^ 4.

Por fim, mesmo uma boa estimativa de concordância, com adequado intervalo de confiança e significância estatística, pode não ser confirmada e aplicada a outras populações, outros avaliadores, outros instrumentos ou a medidas não contidas na amostra primordial, respeitando os princípios inferenciais de generalização da amostra^21^.

## References

[1] Kottner J, Audige L, Brorson S (2011). Guidelines for Reporting Reliability and Agreement Studies (GRRAS) were proposed. J Clin Epidemiol.

[2] Bland JM, Altman DG (1986). Statistical methods for assessing agreement between two methods of clinical measurement. Lancet.

[3] Kuo BI (1994). Intraclass correlation coefficient rather than correlation coefficient to examine agreements among different methods measuring valvular area. Circulation.

[4] Lee KM, Lee J, Chung CY (2012). Pitfalls and important issues in testing reliability using intraclass correlation coefficients in orthopaedic research. Clin Orthop Surg.

[5] Zaki R, Bulgiba A, Ismail R, Ismail NA (2012). Statistical methods used to test for agreement of medical instruments measuring continuous variables in method comparison studies: a systematic review. PLoS One.

[6] Sim J, Wright CC (2005). The kappa statistic in reliability studies: use, interpretation, and sample size requirements. Phys Ther.

[7] Barros FS, Perez JM, Zandonade E (2010). Evaluation of pelvic varicose veins using color Doppler ultrasound: comparison of results obtained with ultrasound of the lower limbs, transvaginal ultrasound, and phlebography. J Vasc Bras.

[8] Fleiss JL, Cohen J (1973). The equivalence of weighted kappa and the intraclass correlation coefficient as measures of reliability. Educ Psychol Meas.

[9] Mandrekar JN (2011). Measures of interrater agreement. J Thorac Oncol.

[10] Brianezi G, Minicucci EM, Marques ME, Miot HA (2013). Evaluation epidermal p53 immunostaining by digital image analysis. Skin Res Technol.

[11] Moura RM, Gonçalves GS, Navarro TP, Britto RR, Dias RC (2011). Transcultural adaptation of VEINES/QOL-Sym questionnaire: evaluation of quality of life and symptoms in chronic venous disease. J Vasc Bras.

[12] Leal FD, Couto RC, Pitta GB (2015). Validation in Brazil of a Questionnaire on Quality of Life in Chronic Venous Disease (Aberdeen Varicose Veins Questionnaire for Brazil/AVVQ-Brazil). J Vasc Bras.

[13] Commenges D, Jacqmin H (1994). The intraclass correlation coefficient: distribution-free definition and test. Biometrics.

[14] Conrad C, Chamlian TR, Ogasowara MS, Pinto MA, Masiero D (2015). Translation into Brazilian Portuguese, cultural adaptation and validation of the Prosthesis Evaluation Questionnaire. J Vasc Bras.

[15] Prieto L, Lamarca R, Casado A, Alonso J (1997). The evaluation of agreement on continuous variables by the intraclass correlation coefficient. J Epidemiol Community Health.

[16] Shrout PE, Fleiss JL (1979). Intraclass correlations: uses in assessing rater reliability. Psychol Bull.

[17] Ianhez M, Fleury LF, Bagatin E, Miot HA (2013). The reliability of counting actinic keratosis. Arch Dermatol Res.

[18] Banerjee M, Capozzoli M, McSweeney L, Sinha D (1999). Beyond kappa: a review of interrater agreement measures. Can J Stat.

[19] Miot HA (2011). Sample size in clinical and experimental trials. J Vasc Bras.

[20] Zou GY (2012). Sample size formulas for estimating intraclass correlation coefficients with precision and assurance. Stat Med.

[21] Donner A, Bull S (1983). Inferences concerning a common intraclass correlation coefficient. Biometrics.

