# Supplementary material for: Análise de concordância em estudos clínicos e experimentais
Source: J Vasc Bras. 2016 Apr-Jun;15(2):89–92. [Article in Portuguese] doi: 10.1590/1677-5449.004216 (PMC5829700; doi:10.1590/1677-5449.004216)
Supplement: Translation

# Translation: Agreement analysis in clinical and experimental trials

**Authors:** Hélio Amante Miot

**Affiliations:** 1 Universidade Estadual Paulista – UNESP, Botucatu Medical School, Department of Dermatology, Botucatu, SP, Brazil.

Analysis of agreement tests the capacity to arrive at identical results (with the same units of measurement), for the same subject/phenomenon, using different instruments, using the same instrument at different times or when performed by different examiners, or some combination of these conditions. Trivial examples include calibration of instruments, testing reliability of scales/measures, assessment of the equivalence of measurement tools, judgment in tests of ability, tests of repeatability or reproducibility, and diagnostic analysis (interpersonal and intraindividual agreement) and psychometric agreement (temporal stability).^1^^,^ 2

It is common for the need to analyze agreement to be, erroneously, met using statistical techniques for measurement of correlation (for example, Pearson’s coefficients), which are only testing the assumption that the variation in the values of one variable follows the variation in the values of another. However, for analysis of agreement, in addition to correlation, there must also be coincidence of values. This is why measures of the effect size of agreement tend to be smaller than correlation coefficients, when applied to the same dataset.^3^^-^ 5

The model of agreement analysis should be defined early on in the project design phase so that a study design is chosen to favor collection, analysis and interpretation of the data. Being able to count on an experienced statistician during this phase will increase the likelihood of success.

In principle, analysis of agreement can depend exclusively on a definition predetermined by the researcher, who should define a tolerance limit that satisfies the research requirements. This is generally the case with calibration and equivalence of measurement instruments, in which measurements must conform to a maximum percentage variation in comparison with a standard measure or a specific instrument. However, the existence of an inherent random error of measurement linked to the instrument and/or the examiners includes an intrinsic variation in measurements, which interferes with estimation of agreement. Several different specific statistical tests have been developed to evaluate these aspects and the most important of them will be discussed below.

The simplest situation occurs when the variable of interest is dichotomous (for example, sick vs. healthy, surgical indication vs. clinical, approved vs. rejected), and estimation is made by two examiners or with two instruments. In such cases, Cohen’s kappa statistic is classically employed. The kappa value, confidence interval and statistical significance should all be interpreted as the magnitude of agreement that exceeds the degree of coincidence of assessments that would occur by chance.^6^

For example, an investigation conducted by Barros et al.^7^ employed lower limb vs. transvaginal Doppler ultrasound for identification of pelvic varicose veins (Table 1), reporting total agreement of (62+93)/249 = 62.2%. The 94 (37.8%) cases of disagreement were distributed in a highly asymmetrical fashion, which revealed a higher rate of diagnostic failure in the lower limb examinations. The kappa coefficient showed weak agreement – 0.31 (95%CI% 0.20-0.40), p < 0.01 – even though it was statistically significant.

**Table 1:** Evidence of pelvic varicose veins according to Doppler ultrasonography of the lower limbs vs. the transvaginal method (n = 249).^7^

**Lower limbs**	**Transvaginal**		**Total**
	**Positive**	**Negative**	
**Positive**	62	6	68
**Negative**	88	93	181
**Total**	150	99	249

A more elaborate situation occurs when an ordinal variable (for example, disease stages, severity levels, graded estimates [0, 1+, 2+, 3+ or 4+, for example], totally correct vs. partially correct vs. error) is estimated by two examiners. In such cases, in addition to total agreement, a weighting can be attributed to similar classifications, favoring them over larger degrees of disagreement. For this type of analysis, the classical choice is the kappa statistic with quadratic weighting (Fleiss-Cohen).^6^^,^ 8

When the same sample is analyzed, the weighted kappa estimator returns a higher magnitude than the measure of complete agreement, since it incorporates the concept of partial agreement. There are several ways of establishing the weights assigned to partial agreements. Usually, kappa with quadratic weightings will return the same result as the Intraclass Correlation Coefficient (ICC), which is discussed below.^8^^,^ 9

For example, using the crude data from the study published by Brianezi et al.,^10^ in which two researchers classified immunohistochemical epidermal marking of the p53 protein using an ordinal scale of 0 to 4+, poor total agreement (16/63=25.4%) between examiners was observed (Table 2). However, the weighted kappa coefficient resulted in substantial agreement – 0.66 (0.40-0.87) – due to the fact that examiner 1 systematically classified images at a higher level than examiner 2, leading to a high degree of partial agreement. Additionally, when there are multiple ordinal levels, classifications at the extreme values (for example, 0 or 4+) tend to result in greater agreement than the intermediate categories.

**Table 2:** Comparative assessment of immunohistochemical epidermal marking (0 to 4+) of p53 protein by two experienced researchers (n = 63).^10^

		**Av2**					**Total**
		**0**	**1+**	**2+**	**3+**	**4+**	
Av1	0	10	-	-	-	-	10
	1+	12	2	-	-	-	14
	2+	7	8	1	-	-	16
	3+	-	6	4	1	-	11
	4+	-	-	3	5	4	12
Total		29	16	8	6	4	63

Av1: evaluator 1; Av2: evaluator 2.

When the variables of interest are quantitative (discrete, continuous or ranks) and two examiners make estimates (interobserver agreement), there are two instruments, or the variables are estimated at different times (test-retest reliability), then the ICC for complete agreement is generally used, which is even robust to violations of normal distribution.^11^^-^ 14 There are a number of different algorithms for calculating ICCs to assess correlation and agreement. However, for the purposes of this text, it is the algorithms for complete agreement that matter. Of these, the researcher must choose between: one-way random, two-way random or two-way mixed, depending on the nature of the examiners. In the first option, the examiners are not the same for each phenomenon assessed; in the second, the examiners are the same for each phenomenon and are chosen at random (the most used); in the third, the examiners are not random, but the only ones possible (for example, intraobserver analysis). Finally, the researcher must choose the single measure ICC if what is important is the agreement of each examiner’s measurement in relation to the behavior of the n examiners (the most used), or the mean measures ICC if the score for the variable is composed of a combination of the n scores of the examiners. These options can result in indicators of different magnitudes.^15^^,^ 16

As an example, the crude data will be used from Ianhez et al.,^17^ in which counts of multiple cutaneous lesions were conducted by two trained examiners – one of them at two different times (A, B1 and B2) – in a study to validate a system for standardized counting of actinic keratoses of the upper limbs (n = 60). The ICC for complete agreement of the intraobserver (B1 vs. B2) comparison (two-way mixed) resulted in 0.74 (0.60-0.84) for single measures and 0.85 (0.75-0.91) for mean measures. However, the ICC for interobserver (A vs. B1) comparison (two-way random) resulted in 0.68 (0.47-0.82) for single measures and 0.81 (0.64-0.90) for mean measures, all with p < 0.01. These results indicate that consistency was greater when a single examiner counted the lesions twice, showing the benefit of using the mean of two measures as an estimate.

Interobserver agreement is usually less than intraobserver agreement for estimates from the same sample, because the first includes sources of variability that are inherent to the different examiners. Additionally, the ICC estimate for single measures generates smaller magnitude estimators than the estimate for mean measures, which justifies the use of multiple measures to reduce the random error.^17^

In addition to careful methodological description of the process of selecting the subjects and the examiners, of the data collection and of the analytical techniques employed, the results of tests of agreement should be expressed in the form of percentage agreement data (total and subsets), in addition to providing the estimators and their 95% confidence intervals and statistical significance. This is the only way that enables interpretation of the circumstances under which variables diverge. The magnitude of estimators of agreement (kappa or ICC) is conventionally interpreted as follows: 0 (absent), 0-0.19 (poor), 0.20-0.39 (weak), 0.30-0.59 (moderate), 0.60-0.79 (substantial), and ≥ 0.80 (almost total).^4^^,^ 6 ^,^ 16

There are generalizations of the algorithms for calculation of the kappa and ICC for multiple assessments and also for different combinations of subjects and examiners. However, these methods are beyond the scope of this text.^1^^,^ 18

Agreement between variables of a quantitative nature can be represented graphically, in pairs, using a Bland-Altman plot, which projects the absolute difference between the measures of each data point on the ordinate axis and the mean of the two measurements on the abscissa axis.^2^ In addition to illustrating the entire distribution, this makes it possible to assess tendencies for agreement to worsen according to the magnitude of the measurements (Figure 1). However, it is not a good measure of the magnitude of agreement. For this purpose the tests of ICC mentioned above are preferable, to complement the graphical representation.

**Figure 1:** Bland-Altman plot of intraobserver counts (B1 x B2) of cutaneous lesions (actinic keratosis) on the upper limbs (n = 60).^17^ Broken line: mean of measurement differences. Dotted line: (95%) interval for distributions of measurement differences.

Returning once more to the crude data collected by Ianhez et al.,^17^ analysis of the plot in Figure 1 reveals that consistency of counts is greater for values below 10 lesions. It is indeed the usual case that agreement suffers an impact from the magnitude of measures. Limitation to a set interval (for example, restricting inclusion to patients with less than 10 lesion) makes the values more reliable in a clinical study.

Choice of the sample size for tests of agreement is dependent on the dimension of the kappa (or ICC), on the test power and on the homogeneity of the categories under analysis. This subject is covered adequately in specialized literature.^6^^,^ 19 ^,^ 20

Indicators of agreement are influenced by the representativeness of each class analyzed, which demands maximum homogeneity of subsets, and also by the modification imposed on the original measurement scale (for example, Log or x^1/n^ transformation). Prior training and control of the rigor of the examiners’ estimates are essential, because their absence could introduce disagreement between estimates, which would add a systematic error, in detriment to the magnitude of the measurements taken.^1^^,^ 4

Finally, even a good estimate of agreement, with an adequate confidence interval and statistical significance, may not be confirmed when applied to other populations, other examiners, other instruments or to measures not contained in the original sample, respecting the inferential principles of generalization of samples.^21^
